# The Evaluation of Emotional Intelligence among Medical Students and Its Links with Non-cognitive Acceptance Measures to Medical School

**DOI:** 10.5041/RMMJ.10365

**Published:** 2019-04-18

**Authors:** Niva Dolev, Nadav Goldental, Ayalla Reuven-Lelong, Tamar Tadmor

**Affiliations:** 1 School of Humanities, Kinneret Academic College, Tzemach, Israel; 2 The Ruth & Bruce Rappaport Faculty of Medicine, Technion–Israel Institute of Technology, Haifa, Israel; 3 Sheba Medical Center, Tel HaShomer, Ramat Gan, Israel; 4 EQ.EL (Emotional Intelligence in Israel Ltd), Haifa, Israel; 5 Hematology Division, Bnai Zion Medical Center, Haifa, Israel

**Keywords:** Emotional intelligence, medical school, medical students, selection, success

## Abstract

**Background:**

The importance of emotional intelligence (EI) to the success of health professionals has been increasingly acknowledged. Concurrently, medical schools have begun integrating non-cognitive measures in candidate selection processes. The question remains whether these newly added processes correctly assess EI skills.

**Objectives:**

Measuring EI levels among medical students; examining the correlations between participants’ EI levels and their scores on the non-cognitive MOR test; and exploring students’ attitudes regarding the importance of EI in medical practice.

**Methods:**

The study included 111 first-year and sixth-year students at the Faculty of Medicine at the Technion, Haifa, Israel. Emotional intelligence was assessed by the Bar-On EQ-i 2.0, and MOR evaluation scores were provided by the faculty. An additional questionnaire was designed to rate students’ attitudes toward the importance of EI to the success of medical doctors (MDs).

**Results:**

No significant correlations were found between MOR test scores and EI evaluation scores. Of the 15 EI competencies evaluated, mean scores for flexibility, problem-solving, and independence were lowest for both the first-year and the sixth-year study groups. No differences in EI levels between first-year and sixth-year students were found. Both groups of students considered EI to be highly important to their success as MDs.

**Conclusions:**

While further studies of the links between MOR tests and EI are required, the current findings indicate that MOR test scores may not be predictive of medical students’ EI levels and vice versa. As previous evidence suggests that EI contributes to professional success and to better outcomes in the field of medicine, integrating it into selection processes for medical students and into the curricula in medical schools is recommended.

## INTRODUCTION

### Emotional Intelligence

Emotional intelligence (EI) deals with the effective integration of emotion and cognition, that is, with the intelligent use of emotions and the use of emotions to improve thought processes.^1^ Emotional intelligence was defined by Salovey and Mayer, both pioneers of EI research, as “the ability to monitor one’s own and others’ feelings and emotions, to discriminate among them and to use this information to guide one’s thinking and actions.”^2^^(p189)^ Using a wider conceptualization, Bar-On defined EI as a set of emotional, personal, and social abilities, skills, and competencies that impact one’s ability to cope with daily demands.^3^ He suggested EI to be composed of five major areas that may contribute to success in life: self-perception, self-expression, interpersonal skills, decision making, and stress management, which are further composed of 15 subscales.^4^ Bar-On’s self-report assessment tool, the EQ-i 2.0, provides a standardized and normalized method for assessing EI levels^4^ and is a widely used EI tool in research and practice.^5^

In the last few decades, as the limited success of cognitive measures to ensure personal and workplace success has been recognized, the concept of EI has been further developed. In particular, social and emotional skills were found to play an important role alongside cognitive skills,^6^ to be inseparable from cognitive processes, and to be crucial for effective thought and decision-making processes.^2^ Indeed, many studies have linked EI to physical and psychological health,^3^ stress coping and lower burnout rates,^7^ professional success,^8^–^10^ effective teamwork, and to enhanced leadership skills,^11^ across a variety of professions.^3^ In the medical arena too, it has been noted that EI plays an important role in the professional success of medical doctors (MDs),^12^ including doctor–patient relations, patient satisfaction, accurate diagnosis, adherence to treatment protocols, decrease in lawsuits, teamwork, stress-coping skills, organizational commitment, and medical leadership.^13^–^23^ Additionally, academic success among medical students has also been linked to higher EI levels.^24^,^25^

More particularly, EI appears to be crucial to success when faced with the constantly changing and increasingly challenging reality in the field of medicine in the twenty-first century. With increasing lifespans, fast-changing technologies, more demanding patients and families, and the multi-generational nature of health organizations, MDs are now facing greater workloads, increasing pressures and demands, more regulation, and a constant need to keep current with innovations, and are simultaneously called upon to provide better care, to learn constantly, to engage in research, and to lead.^26^,^27^

Emotional intelligence skills, such as awareness of self and others, communication skills and empathy, impulse control, stress tolerance, flexibility, and the capacity to maintain optimism, are increasingly required for coping with challenges and for succeeding in the medical professions.^13^,^14^ Consequently, it has been increasingly suggested that EI measures should be included as part of admittance processes in medical schools^25^ and that EI development should be included in medical studies curricula.^27^–^35^

So far, however, such processes have not yet been integrated into general practice in medical schools in Israel.

### Non-cognitive Assessments for Medical School Candidates

With the growing understanding that cognitive and professional skills, which have long provided the main admission criteria for medical schools, are insufficient for identifying the best candidates for medical studies, medical schools in Israel and elsewhere have started employing assessments designed to evaluate non-cognitive skills and ethical attitudes. For example, the Multiple Mini-Interviews (MMIs) tool, now widely used in the USA, provides unique information regarding candidates’ personal and interpersonal attributes.^36^–^38^

Following these attempts, some medical schools in Israel, including the Faculty of Medicine at the Technion, Haifa, started integrating non-cognitive skill assessments, and in particular the MOR test (the Hebrew acronym for “Selection for Medicine”), into their candidate evaluation processes.

The MOR test comprises several parts, including an eight-station behavioral assessment tool, where candidates encounter simulated patients and join in group tasks. Candidate performance is evaluated by a number of trained raters, all medical school faculty, acting as both interviewers and viewers. This process is supplemented by an autobiographical questionnaire, and by a judgement and decision-making questionnaire.^38^,^39^

These tools were designed to examine candidates’ ethical behaviors and attitudes, manner of coping with dilemmas and with ambiguous situations, and levels of empathy, reflective ability, and the ability to identify emotions.^36^–^39^ More generally, candidates are evaluated with respect to four qualities: interpersonal communication skills; stress-coping abilities; initiative and responsibility; and self-awareness.^38^ Many of these skills are included in the framework of EI.

However, despite such similarities between MOR and EI evaluations, and while EI skills have been noted as important to the professional and ethical success of MDs,^13^,^14^ the two measures have been scarcely studied simultaneously or compared.^40^,^41^ More specifically, the degree to which MOR tests assess EI skills has not yet been established.

Furthermore, while EI skills have been shown to be learnable, and while recent publications underscore the need to develop EI in medical students,^25^–^33^ the incorporation of EI skill development programs into medical schools in Israel and the attitudes of students toward such a potential change in the schools’ curricula have not yet been examined.

Accordingly, the goals of the current research were: to examine general EI levels and specific EI skills in first-year and sixth-year medical students at the Faculty of Medicine at the Technion, Haifa, Israel; to identify differences in EI levels between these two student groups; to examine correlations between EQ-i scores and MOR scores among all participant students; and to examine students’ attitudes regarding the relevance of EI to MDs’ work and toward the incorporation of EI into the curricula of medical studies.

## METHODS

An observational, cross-sectional design was employed. Participants included 111 students of medicine: 58 first-year students and 53 sixth-year students, from the Rappaport Faculty of Medicine at the Technion, Israel Institute of Technology, in Haifa, Israel (Table 1). The two groups of students were selected to represent the beginning (first year) and the end (sixth year) of the study program. In each group, selection of participants was based on availability. The samples represent 45% and 50% of registered students at the time of the study in each of these two groups, respectively. Such a response rate is relatively high given the nature of the study (a survey, as opposed to a clinical trial) and given the study population of medical students where even a 20% response rate is considered adequate.^21^,^40^ For each participant we obtained demographic data, as well as scores for three measures: EI, MOR, and an Attitudes toward EI in Medical Settings questionnaire.

**Table 1:** Study Population.

Student Characteristics	First Year (*n*)	Sixth Year (*n*)
Total No. Students	58	53
Jewish Students	28	21
Arab Students	27	32
Unknown Ethnicity	3	0
Male	20	21
Female	32	32
Unknown Gender	6	0
Having MOR Scores	30	24

The study was approved by the Helsinki committee in the Bnai Zion Medical Center in Haifa, and prior permission to conduct the study was received from both the management and the Students’ Association at the Technion. Additionally, individual consent forms were obtained from each of the participating students, including permission to access and review individual MOR scores. In addressing the various parties, the voluntary and anonymous nature of the study was emphasized.

### Research Tools

#### Emotional intelligence

The Bar-On EQ-i 2.0 assessment tool^4^ was used to evaluate the 5 main EI areas (and the 15 corresponding subscales) that constitute the Bar-On model of EI: Self-perception (emotional self-awareness, self-regard, and self-actualization); Self-expression (emotional self-expression, assertiveness, and independence); Interpersonal skills (interpersonal relations, empathy, and social responsibility); Decision making (reality testing, problem solving, and impulse control); and Stress management (stress tolerance, optimism, and flexibility)—as well as a well-being indicator.

This self-report tool includes 133 statements rated on a five-point Likert scale (1, “not true of me” or “hardly true”, to 5, “always or very often true of me”). Reliability and validity of the measure have been examined in large and diverse populations, including in Israel.^4^ The average predictive validity coefficient was found to be 0.59, and the measure was found to differentiate between individuals and to correctly identify changes following EI training.^4^

Raw scores are processed and tabulated by the test publisher (MHS) and are converted to standard scores based on an average score of 100 with a SD of 15. These include mean general EI scores, as well as mean scores for the 5 main EI areas and for the 15 corresponding subscales based on an extensive normative base of Western population.^39^

#### MOR tests

The MOR test, a modification of the internationally employed MMI test, is the common screening test for medical school applicants in Israel and is routinely used by the Technion School of Medicine for that purpose. Composite scores consist of two independently calculated scores: One combines scores for two questionnaires (an autobiographical one, and a judgement and decision-making one), and the other for behavior assessment tasks. The two scores are weighted at 30% and 70%, respectively, and are combined to provide a composite score using a conversion formula. The calculated average MOR score is 200 with an SD of 20, and the range of scores has been found to be 150–250.^39^ General composite MOR scores are provided by Israel’s National Institute for Testing and Evaluation.^39^

The scores for all students participating in the current study were provided to the authors by the Faculty of Medicine in the Technion, Haifa, after receiving the participants’ consent. In agreement with the measure’s specifications, the scores for the participants ranged from 150 to 250, with an average of 200.

#### Attitudes toward EI in medical settings questionnaire

This multiple-choice questionnaire was constructed especially for the present study in order to examine students’ perceptions of the importance of various skills, and in particular EI skills, to the work of MDs.

The items selected for this questionnaire were based on previously published studies as well as on interviews with six MDs who served both as hospital staff and as members of the Faculty of Medicine at the Technion in Haifa.

An inter-rater reliability procedure was utilized to assure content validity. As part of this procedure, analysis of the proposed items was carried out by two raters, both of them MDs and members of the Faculty of Medicine at the Technion and both experienced in the field of emotional intelligence in medicine. Inter-rater Cohen’s Kappa (k) reliability,^40^ commonly employed in psychological research, was used. Following the above procedure, relevant skills were selected based on their content validity, and the participant students were asked to rate the importance of these skills on a five-point Likert-type scale (1, “little importance”, to 5, “very important”).

### Data Analysis

Statistical analysis was conducted using an SPSS 23 program. Differences in mean MOR scores, EI scores, and in attitudes between first-year and sixth-year students were calculated using *t* tests. Non-linear correlations were used to identify trends and correlations between factors, and Pearson coefficients were indicated to identify trends. In order to identify correlations between EI scores and the importance students attributed to the different EI skills, *Z* values were calculated with *P*<0.05.

## RESULTS

### Study Population

Of the 111 medical student participants (58 first-year students and 53 sixth-year students), 41 were males and 64 were females. In terms of ethnicity, 49 were Jews and 59 were either Muslim or Christian Arabs (Table 1).

### Emotional Intelligence Scores

Descriptive statistics, indicating the degree of difference between mean scores for the study group and for the general population, revealed that the mean general EI scores for first-year and sixth-year student participants (Table 2) were 96.84 and 93.21, respectively (with an assigned mean value of 100 for the general population).

**Table 2:** Emotional Intelligence Skills for First- and Sixth-year Students.

Emotional Intelligence Skill	No. Participants		Average		SD		Diff.	Signif.

	First Year	Sixth Year	First Year	Sixth Year	First Year	Sixth Year		
**General EI**	58	53	96.84	93.21	10.614	10.496	3.637	0.073

**Self-perception**	58	53	101.55	98.91	12.131	11.694	2.646	0.245
Self-regard	58	53	101.09	97.43	14.828	16.214	3.652	0.218
Self-actualization	58	53	102.81	99.13	15.283	12.824	3.678	0.175
Emotional Self-awareness	58	53	102.64	103.58	10.199	10.730	−0.947	0.635

**Self-expression**	58	53	95.66	91.49	11.279	14.114	4.165	0.093
Emotional Expression	58	53	96.55	97.00	15.685	12.319	1.118	0.708
Independence	58	53	93.05	88.49	11.041	15.220	4.561	0.072
Assertiveness	58	53	101.81	97.00	10.470	12.139	4.810	0.028

**Interpersonal**	58	53	103.83	104.15	12.713	11.758	−0.323	0.890
Interpersonal Relationships	58	53	100.41	98.58	13.277	12.036	1.829	0.448
Empathy	58	53	105.31	108.34	11.970	11.579	−3.029	0.179
Social Responsibility	58	52	103.48	102.81	13.723	12.373	0.675	0.788

**Decision Making**	58	53	94.52	91.09	11.257	12.180	−0.323	0.890
Problem Solving	58	53	90.98	83.25	10.673	15.390	7.737	0.002
Reality Testing	58	53	101.14	101.98	11.727	10.252	−0.843	0.687
Impulse Control	58	53	96.52	95.96	12.521	15.148	0.555	0.835

**Stress Management**	58	53	91.84	86.91	10.508	12.937	4.939	0.029
Flexibility	58	53	86.09	83.23	12.072	14.491	2.860	0.260
Stress Tolerance	58	53	99.19	93.87	10.572	12.400	5.322	0.016
Optimism	58	53	95.34	91.04	15.782	15.240	4.307	0.147

Diff, difference; Signif., significant

Of the five main areas of EI, scores for both the first-year and sixth-year students were lowest for: stress management (91.84±10.508 and 86.91±12.937, respectively), decision making (94.52±11.257 and 91.09±12.180, respectively), and self-expression (95.66±11.729 and 91.49±14.114, respectively). Both groups showed highest scores on the interpersonal composite scale and lowest on the stress management composite scale.

Assessment of the 15 subscales constituting the 5 main EI areas (Table 2) revealed that mean scores for both the first-year and the sixth-year groups were highest for empathy (105.31±11.970 and 108.34± 11.579, respectively) and for social responsibility (103.48±13.723 and 102.81±12.373, respectively), both Interpersonal skills. The first-year group also scored highly on self-actualization (102.81±15.283), while the sixth-year group scored highly on self-awareness (103.58±10.730). The lowest average scores for both the first-year group and the sixth-year group were noted for flexibility (86.09±12.072 and 83.23± 14.491, respectively), problem solving (90.98± 10.673 and 83.25±15.390, respectively), and independence (93.05±11.041 and 88.49±15.220, respectively).

As seen in Table 2, the mean general EI score for the first-year student group was significantly higher than that for the sixth-year student group (96.84± 10.614 and 93.21±10.496, respectively). Among the five main EI areas, a significant difference between the two groups was found in stress management (91.84±10.508 and 86.91±12.937, respectively).

Additionally, significant differences between the two groups were found in 3 of the 15 EI subscales: problem solving (a 7.737-point difference), stress tolerance (a 5.322-point difference), and assertiveness (a 4.81-point difference) (*P*<0.05). In all cases, scores for the first-year group were higher than those for the sixth-year group.

### MOR Scores

Mean MOR scores for the first-year student group and for the sixth-year student group were 205.70 and 208.96, respectively (Table 3).

**Table 3:** MOR Scores.

	First Year	Sixth Year
Participants (*n*)	30	24
Average Score	205.70	208.96
SD	5.724	8.705

### Correlations between EQ-i Scores and MOR Scores

Examination of MOR scores versus both mean general EI scores and scores for the five main EI areas, among both the first-year and the sixth-year student groups, did not reveal any significant correlations.

With regard to the 15 EI subscales, a significant (*P*<0.05) correlation was noted between MOR scores and emotional self-awareness, self-regard, independence, and stress tolerance for the sixth-year student group. No such correlations were found for the first-year student group (Table 4).

**Table 4:** Correlation Between EI Composites and MOR Scores.

El Dimension/Dependent Variable	Adjusted R Square		F (ANOVA		Significance (ANOVA)	
	First Year	Sixth Year	First Year	Sixth Year	First Year	Sixth Year
**Self-perception**						
Self-regard	−0.020	0.407	0.820	6.267	0.495	0.004*
Self-actualization	0.071	0.131	1.683	1.990	0.197	0.125
Emotional Self-awareness	−0.013	0.303	0.883	4.340	0.464	0.016†
**Self-expression**						
Emotional Expression	−0.114	−0.039	0.760	0.711	0.972	0.557
Assertiveness	0.100	0.173	1.997	2.606	0.141	0.080
Independence	−0.055	0.230	0.530	3.294	0.666	0.042†
**Interpersonal**						
Interpersonal Relationships	−0.077	0.025	0.358	1.200	0.783	0.335
Empathy	0.063	0.085	1.605	1.714	0.214	0.196
Social Responsibility	−0.022	−0.027	0.810	0.805	0.501	0.507
**Decision Making**						
Problem Solving	0.114	0.203	2.153	2.952	0.120	0.057*
Reality Testing	−0.015	0.093	0.868	1.789	0.471	0.182
Impulse Control	−0.051	0.043	0.564	1.346	0.644	0.288
**Stress Management**						
Flexibility	0.052	0.153	1.497	2.383	0.241	0.100
Stress Tolerance	0.055	0.380	1.523	5.702	0.234	0.005*
Optimism	−0.097	--	0.205	--	0.892	--

* Correlation is significant at the 0.01 level (two-tailed).

† Correlation is significant at the 0.05 level (two-tailed).

### Attitudes toward EI

All students assigned high levels of importance to EI skills, rating most of them at 4 and above on an importance scale of 1–5 (Table 5). The five skills that were ranked highest in importance by both the first-year and the sixth-year groups, and the corresponding mean score values, respectively, for each group, were: ethical conduct (4.79±0.411 and 4.68±0.51), problem-solving (4.67±0.546 and 4.74±0.445), learning ability (4.7±0.499 and 4.7±0.503), stress tolerance (4.67±0.557 and 4.49±0.639), interpersonal relations (4.4±0.708 and 4.55±0.539 and), and empathy (4.54±0.629 and 4.55±0.667 and). No significant differences between the two groups were noted for those skills. Medical technical skills were ranked lowest by both student groups (3.65±0.790 and 3.49±0.933 and, respectively).

**Table 5:** The Importance Assigned by Students to EI Skills: Average and SD.

El Skill	No. Students		Average		SD	
	First Year	Sixth Year	First Year	Sixth Year	First Year	Sixth Year
Self-regard	56	53	4.13	4.09	0.605	0.766
Self-actualization	56	53	4.14	4.04	0.724	0.831
Emotional Self Awareness	57	52	4.33	4.48	0.636	0.577
Assertiveness	57	53	4.32	4.28	0.572	0.632
Independence	57	53	4.14	4.06	0.600	0.781
Interpersonal Relationships	57	53	4.44	4.55	0.708	0.539
Empathy	57	53	4.54	4.55	0.629	0.667
Social Responsibility	56	53	4.20	4.11	0.724	0.847
Problem Solving	57	53	4.67	4.74	0.546	0.445
Reality Testing	57	53	4.42	4.47	0.596	0.608
Flexibility	56	53	4.18	4.28	0.664	0.690
Stress Tolerance	57	53	4.67	4.49	0.577	0.639
Optimism	57	53	4.02	3.68	0.790	0.779
Happiness	57	53	3.75	3.55	0.830	0.889
Analytical Skill	56	52	4.32	4.44	0.636	0.574
Patience	57	53	4.63	4.55	0.587	0.667
Medical Technical Skills	57	53	3.65	3.49	0.790	0.933
Tolerance to Others	57	53	4.44	4.28	0.655	0.744
Quick Response	57	53	4.42	4.08	0.706	0.851
Learning Ability	57	53	4.70	4.70	0.499	0.503
Accuracy	57	53	4.42	4.21	0.625	0.661
Ethical Conduct	57	53	4.79	4.68	0.411	0.510
Self-expression	57	53	4.05	4.25	0.639	0.677
Spatial Perception	56	53	3.66	3.66	0.837	1.058
Deductive Reasoning Skills	56	53	4.05	4.19	0.616	0.681

Significant differences between the first-year and sixth-year groups, respectively, were for optimism (4.02±0.79 and 3.68±0.779,) and quick response (4.42±0.706 and 4.08± 0.851,) respectively. Furthermore, the first-year group considered these skills more important to MDs’ work than did the sixth-year group (Table 5).

## DISCUSSION

The purpose of the current study was to determine if correlations exist between the scores for the MOR test, a non-cognitive admission test for medical studies, and the emotional intelligence of medical students (as measured by the EQ-i 2.0 assessment tool). This topic has been gaining added relevance given the fact that medical schools are regularly tasked with selecting the next generation of MDs among an increasing number of candidates, and devote considerable amounts of resources to ensure that only the most competent and ethical candidates are selected.

To date, most screening processes for future MDs have relied on cognitive measures, suggesting that medical programs assign higher importance to cognitive abilities as compared with non-cognitive skills. Thus, medical schools may miss good potential candidates while selecting others who may not own the full range of skills required from MDs.^21^ Recently, with the growing understanding that non-cognitive skills play a major role in MDs’ success,^13^–^23^ there is a growing global attempt, including in Israel, to identify candidates who demonstrate superior non-cognitive skills in addition to the highest cognitive abilities. For that purpose, dedicated tools for assessing non-cognitive skills are increasingly employed. In Israel, the locally designed non-cognitive tool of choice in most medical schools is the MOR test. However, this composite evaluation tool has not yet been validated by faculties of medicine across Israel, and the exact non-cognitive skills it measures have not yet been clearly identified. Furthermore, while emotional intelligence has been recognized as a significant contributor to the success of MDs in the twenty-first century, links between EI and currently used non-cognitive admittance measures to medical schools, such as the MOR test, have not yet been tested.

To attend to this gap in knowledge, the current study, conducted among first-year and sixth-year medical students in the Technion, Haifa, compared the EQ-i 2.0 measure of EI to the MOR non-cognitive assessment tool used by the Technion.

The present results indicate the absence of any correlations between the five main EI scores and the MOR scores (*P*<0.05). These findings raise some doubts with respect to the ability of medical programs that employ the MOR test to select candidates for the full range of skills required for MDs, and in particular EI skills, which were previously noted as important assets for MDs. Such doubts are in line with a study by Boden et al. who discussed screening procedures for residency positions and wondered whether a non-cognitive tool that does not correlate with EI may be inadvertently selecting future physicians with diminished soft skills. This, they claimed, could have implications in areas such as patient satisfaction and quality of medical treatment.^21^

Comparison of MOR results and mean scores for the 15 EI subscales did reveal significant (*P*<0.05) correlations between MOR scores and several skills: self-awareness, self-regard, independence, and stress tolerance, among the sixth-year student group. No correlations were found between MOR scores and any of the 15 EI subscales among the first-year student participants.

These findings, while preliminary, may suggest that the MOR test is designed to be more sensitive to certain skills rather than others. Based on the available data, it is unclear whether the MOR targets these specific skills intentionally.

Yet, even if this is the case, a range of additional skills, such as impulse control, empathy, or maintaining a positive outlook, are similarly important for the medical work and need to be similarly assessed.

It is important to note that previous studies have examined links between admission scores on a variety of admission tests and EI scores in medical school candidates,^36^ and some of these studies similarly have not found any correlation between the two sets of scores.^41^ Others found positive correlations between interview scores and scores of specifically designed EI evaluation tools.^36^ However, these last-mentioned studies did not examine the MOR specifically and did not use a general, validated, and widely used measure of EI, but rather a scale specifically designed for the study In particular, previous studies did not use an EI scale that captures a wide set of EI skills such as the EQ-i.

Furthermore, in both the first-year and sixth-year groups in the current study the levels of many EI skills were not higher than those for the general population. These findings may serve as an indication that the MOR test does not assess students’ EI skills and does not favor emotionally intelligent candidates. Given the complex work MDs are required to perform, involving minute-by-minute decisions and problem solving, high stress, constant interactions in emotionally charged situations, and the need to work in teams, higher than average EI skills are required.

Some previous transnational studies of medical students have indicated EI scores similar to, or slightly lower than those of the general population.^37^–^39^ A possible explanation may be that medical students tend to focus on cognitive learning both before entering medical school and during medical studies, and that cognitive skills have been the main measure of success in medical schools. For example, lower than average EI scores were noted among gifted students in a study where a self-report measure, similar to the method used in the current study, was employed.^42^ These findings, combined with the present results, further highlight the need to both assess and cultivate EI skills in medical students.

Also of note is the fact that sixth-year students who had participated in the present study did not score higher than their first-year counterparts on general EI, or on any of the main EI areas or subscales. The small decline in scores that was noted for several EI skills among sixth year students, as compared with their first-year counterparts, was not significant, and the current results are consistent with a zero change in EI over the study period of six years. While some earlier studies found sixth-year students to score higher than first-year students in specific EI skills, such as self-regulation,^43^,^44^ the current findings do not support such findings. Indeed, other studies found no such increase or a decline in EI levels over the course of medical studies.^38^,^39^,^45^,^46^

A possible explanation for the current findings may be a prevailing focus on cognitive skills, and possibly a lack of support or reinforcement of social-emotional skills, in medical schools. Furthermore, medical studies are highly demanding and challenging. For many, the challenge of balancing between personal happiness, social responsibility, and the demands of professional (or clinical) training, as well as the inevitable exposure to illness and suffering, can be overwhelming and stressful. Stress, in turn, was noted to have the potential to reduce emotional resources and thus EI.^47^,^48^

In particular, EI was found to decrease with increase in total workload. In medical schools, the number of night shifts and emergency duty hours, and therefore the workload, all increase over the course of the medical studies period, and thus an increase in stress levels and a decrease in EI among medical students are a possible outcome.^44^

If indeed EI levels do not improve with increased years-of-attendance in medical school, then clearly this is a cause for worry, as numerous studies have shown EI to be important to the work of MDs.^12^–^25^ One would wish for medical students to constantly improve their EI skills in the course of their studies, as professional challenges and demands are likely to increase with the advancement of their careers, and with them the need to use EI skills is similarly likely to increase. Yet the above cited studies suggest that workloads and other experiences in the medical school environment may in fact decrease EI levels. It follows that programs that could incrementally and methodically enhance EI levels among medical students should be included in medical school curricula.^30^,^31^ Studies have demonstrated the ability to enhance EI through training in higher-education settings.^49^,^50^ Such training programs may include designated workshops and a variety of EI-related subjects, and the relevant themes could be further augmented and supported by faculty staff and other mentors. The noted importance assigned by participant medical students to EI skills suggests that such integration may be welcomed and could provide a window of opportunity for the inclusion of EI training programs in the curricula of medical schools.

### Research Limitations

Firstly, the study was conducted in a single medical school in Israel and may not represent other schools, in Israel or elsewhere.

In terms of the study population, participants included only first-year and sixth-year students in the Faculty of Medicine at the Technion, Haifa, and only a sample from each group. However, first-year and sixth-year students represent two significant stages in the study program, namely the beginning and the end stages, and samples within each of these two groups were relatively large. Furthermore, both groups attended the same medical school and have undergone very similar admission procedures, lending some support to comparisons between the two groups and to interpretations of changes in EI values as a function of the years-in-school parameter.

It is also important to note that as the methodology employed in the present study is consistent with a survey, rather than with a clinical trial, the sample achieved, of almost 50% of registered students for each cohort, was larger than response rates noted previously in similar studies.^22^,^38^

Furthermore, EI was evaluated using a single framework and a single corresponding tool, those of Bar-On. Similarly, only a single non-cognitive admission test, the MOR, was examined. Additional studies may examine the links between EI and non-cognitive admission tests for medical schools using a variety of EI tools, and may do so across a variety of stages in the study program, in a variety of schools and countries. A longitudinal study may help clarify the changes in EI over the course of medical studies. Additionally, an interventional study, whereby some of the participants would receive personal training aimed at developing their emotional intelligence abilities, should be considered.

## CONCLUSION

In summary, this study of emotional intelligence in medical students is preliminary, and findings require further validation through additional studies. However, and despite the noted research limitations, the present study offers a unique perspective of assessment methods of abilities and skills of medical students and highlights the importance of further exploration of these methods, as well as of selection processes and training programs for future MDs. Such added knowledge is necessary in order to allow MDs to have the skills most important for providing outstanding medical care in the twenty-first century.

## Abbreviations

EI: emotional intelligence
MD: medical doctor.
